# Electronic Health Record Use in Swiss Nursing Homes and Its Association With Implicit Rationing of Nursing Care Documentation: Multicenter Cross-sectional Survey Study

**DOI:** 10.2196/22974

**Published:** 2021-03-02

**Authors:** Dietmar Ausserhofer, Lauriane Favez, Michael Simon, Franziska Zúñiga

**Affiliations:** 1 College of Health Care-Professions Claudiana Bolzano-Bozen Italy; 2 Nursing Science Department of Public Health University of Basel Basel Switzerland; 3 Nursing Research Unit Inselspital Bern University Hospital Bern Switzerland

**Keywords:** electronic health records, nursing homes, nursing care, health care rationing, rationing of nursing care, unfinished care, documentation, patient care planning, mobile phone

## Abstract

**Background:**

Nursing homes (NHs) are increasingly implementing electronic health records (EHRs); however, little information is available on EHR use in NH settings. It remains unclear how care workers perceive its safety, quality, and efficiency, and whether EHR use might ease the burden of documentation, thereby reducing its implicit rationing.

**Objective:**

This study aims to describe nurses’ perceptions regarding the usefulness of the EHR system and whether sufficient numbers of computers are available in Swiss NHs, and to explore the system’s association with implicit rationing of nursing care documentation.

**Methods:**

This was a multicenter cross-sectional study using survey data from the Swiss Nursing Homes Human Resources Project 2018. It includes a convenience sample of 107 NHs, 302 care units, and 1975 care workers (ie, registered nurses and licensed practical nurses) from Switzerland’s German- and French-speaking regions. Care workers completed questionnaires assessing the level of implicit rationing of nursing care documentation, their perceptions of the EHR system’s usefulness and of how sufficient the number of available computers was, staffing and resource adequacy, leadership ability, and teamwork and safety climate. For analysis, we applied generalized linear mixed models, including individual-level nurse survey data and data on unit and facility characteristics.

**Results:**

Overall, the care workers perceived the EHR systems as useful; ratings ranged from 69.42% (1362/1962; *guarantees safe care and treatment*) to 78.32% (1535/1960; *allows quick access to relevant information on the residents*). However, less than half (914/1961, 46.61%) of the care workers reported sufficient computers on their unit to allow timely documentation. Half of the care workers responded that they sometimes or often had to ration the documentation of care. After adjusting for work environment factors and safety and teamwork climate, both higher care worker ratings of the EHR system’s usefulness (β=−.12; 95% CI −0.17 to −0.06) and sufficient numbers of computers (β=−.09; 95% CI −0.12 to −0.06) were consistently associated with lower implicit rationing of nursing care documentation.

**Conclusions:**

Both the usefulness of the EHR system and the number of computers available were important explanatory factors for care workers leaving care activities (eg, developing or updating nursing care plans) unfinished. NH managers should carefully select and implement their information technology infrastructure with greater involvement and attention to the needs of their care workers and residents. Further research is needed to develop and implement user-friendly information technology infrastructure in NHs and to evaluate their impact on care processes as well as resident and care worker outcomes.

## Introduction

### Background

Health care organizations worldwide are increasingly using electronic health records (EHRs) to improve health care safety, quality, and efficiency. EHRs are defined as an electronic version of a person’s medical history, including key administrative clinical data relevant to that person’s care [1]. Although digital transformation in acute care is progressing quickly, the implementation of EHR in long-term care is following at a slower pace. In the United States, less than 50% of nursing homes (NHs) have implemented EHRs, with nonprofit and government NHs, those with more than 100 beds, and those with higher staffing levels (ie, registered nurses [RNs] and certified nursing assistants) more likely to use EHRs [2-6]. Among the barriers identified for successful EHR implementation, NH settings were costs, the need for training, and the culture change required to embrace technology [6,7].

Although little is known regarding the impact of EHR adoption on the provision of NH care, positive effects on the processes and outcomes of acute care provision have been reported. These include increased adherence to guideline-based care, enhanced surveillance and monitoring, improved clinical decision making, and decreased medication errors [8-13]. Despite concerns that EHR implementation might negatively impact safety and quality of care during the transition period, acute care studies found no differences between pre- and postimplementation on short-term inpatient mortality, adverse events, or readmissions [14]. Some benefits of EHR use (eg, increased access to resident information, cost avoidance, and increased documentation accuracy) are increasingly recognized by health care professionals, including physicians [15] and nurses [16].

Even if the overall quality of documentation is not improved in the electronic system, for example, in cases where paper-based documentation standards were already extremely high [17], one expected benefit of EHR is increased time efficiency. In fact, at least during the implementation phase, the opposite has been reported, with documentation time increasing from 16% to 28% for physicians and from 9% to 23% for nurses [18]. Although EHRs should support health care professionals by reducing their documentation burden, thus allowing them more time for dedicated patient care, this initial impact on their workloads might prove a major barrier to their implementation and long-term use [18].

Nurses spend around one-fifth of their working time on documentation activities, such as developing or updating nursing care plans [19]. Although these activities are considered crucial to the provision of high-quality professional NH care [20], these *indirect care* activities performed away from residents are often either rationed or missed. Nurses place higher priority on direct care activities, that is, those that require interactions with the residents or their families, such as assisting with drinking and food intake [21,22]. A previous study reported that NH care workers who reported less rationing of direct care, rehabilitation, monitoring, and social care activities tended to perceive the overall quality of NH care as higher, whereas they actually associated *more* rationing of documentation with better self-perceived quality of NH care [23].

**Figure 1:**
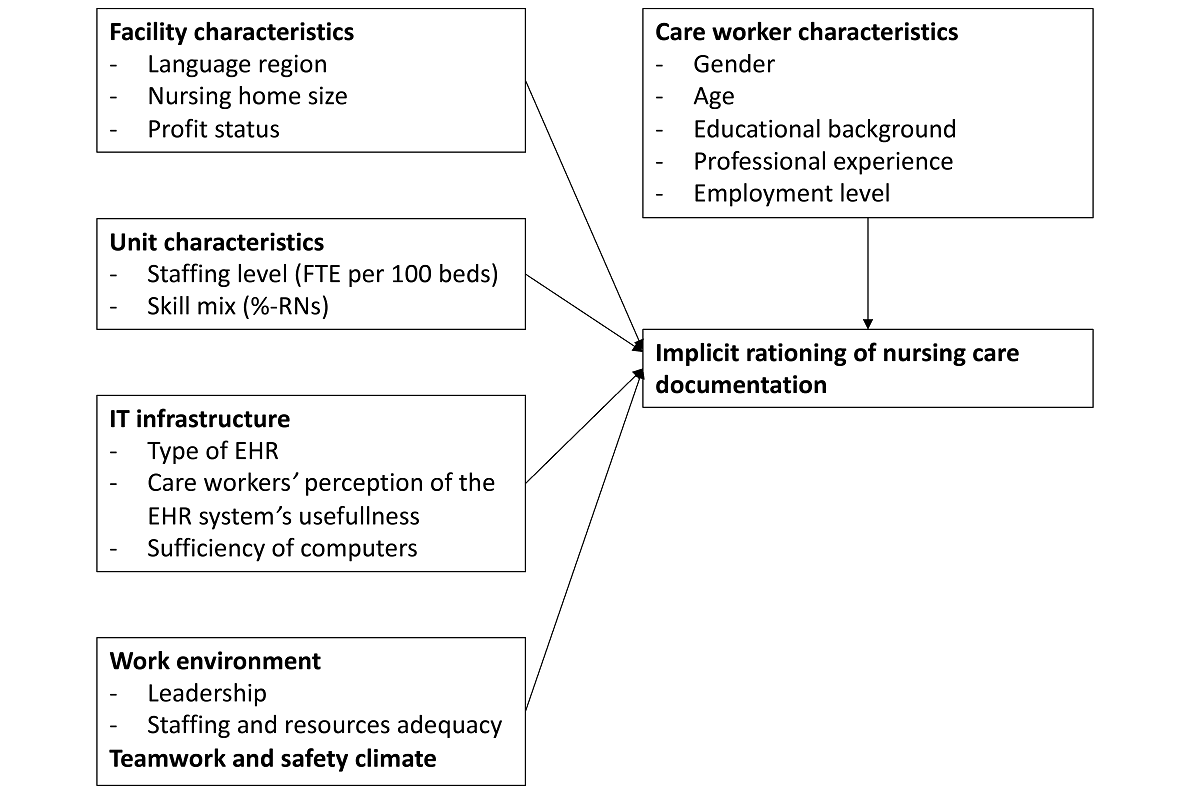
Conceptual framework: factors related to implicit rationing of nursing care documentation. EHR: electronic health records; FTE: full-time equivalent; RN: registered nurse.

*Implicit rationing of nursing care or missed care—recently summarized also under the umbrella term unfinished nursing care* [24]—has become a global phenomenon of concern affecting the safety and quality of hospital and NH care [25,26]. NH studies indicate that up to 75% of nurses leave at least one necessary care activity unfinished on every shift [22,27]. Implicit rationing of nursing care has been defined as “the withholding of or failure to carry out all needed nursing interventions in the face of inadequate time, staffing or skill mix” [28]. Although this mainly refers to *direct care* activities with residents, failure to document nursing care is equally dangerous, as it hinders continuity of care. As this study’s conceptual model describes (Figure 1), alongside perceived shortfalls in the information technology (IT) infrastructure (ie, EHRs and computers), care workers’ perceptions of facility and unit characteristics, work environment, teamwork and safety climate, and even individual care worker characteristics can all impact NH care provision processes, meaning they can also result in implicit rationing of nursing care, including documentation. Evidence supports this conceptual underpinning, as lower levels of nurse staffing [29] and teamwork and safety climate [21] were all associated with higher amounts of missed or rationed care.

### Research Gap and Objectives

To date, little information is available on EHR use in NHs, for example, how nurses, as the main users, perceive their workplace system’s quality and efficiency. Moreover, it remains unclear what roles EHRs’ uses and characteristics might have on NH care processes, for example, whether more efficient EHRs might reduce care workers’ documentation burden, thereby reducing the perceived need to implicitly ration it and allowing better continuity of care. As increasing numbers of NHs have implemented EHRs in recent years with the objective of increasing efficiency, in this study, we aim (1) to explore Swiss NH care workers’ perceptions regarding their EHR systems’ usefulness and the sufficiency of the number of computers and (2) to explore the association between the IT infrastructure and implicit rationing of nursing care documentation.

## Methods

### Study Design

This study is based on data from the 2018 Swiss Nursing Home Human Resources Project (SHURP), a cross-sectional, multicenter study.

### Sample and Setting

A convenience sample of 107 NHs, housing 302 care units, and 1975 care workers (ie, RNs and licensed practical nurses) in Switzerland’s German- and French-speaking regions were included in this study. The mean response rate to the care worker survey was 66.0%, ranging from 12.7% to 98.2% at the facility level. NHs who had participated in the first edition of the SHURP study (2013-2015) [30] were invited to participate in this new edition and were automatically included if they accepted. To increase the sample size, we sent waves of invitations to randomly selected NHs. In parallel, uninvited NHs that were willing to participate could contact the study team directly to be included. Finally, to further increase the inclusion rate, collaborations were set up with diverse NH associations. Additional NHs were included until March 2019. Inclusion criteria were that each NH was recognized by cantonal authorities and had a minimum of 20 beds.

### Data Collection

The survey was administered, as appropriate, in two language versions, German and French, between September 2018 and October 2019. All directors of the participating NHs provided written consent to participate in the study. For care workers, sending back the voluntary care worker questionnaire was considered as informed consent.

### Ethical Aspects

An ethics waiver was obtained from the responsible Swiss ethics committee (the Northwest and Central Switzerland ethics committee, BASEC Nr Req-2018-00420).

### Variables and Measures

To measure *the rationing of nursing care documentation*, we used the 3-item subscale of the NH version of the Basel Extent of Rationing of Nursing Care instrument. Care workers were asked how often in the past 7 days they had been unable to study care plans at the beginning of their shift, set up or update residents’ care plans, or document the care provided because of lack of time or high workload [31]. As lack of time or workload is a matter not only of resources (eg, staffing levels) but also of demand, EHR systems might increase the demand in terms of documentation.

The main explanatory variables were *care workers’ perceptions of the EHR system’s usefulness* (5 items) and *sufficiency of the number of computers on the units* (one item). These items were developed based on a literature review of EHR use in NHs [32,33]. The explanatory factor analyses of the internal structure of the 5 items on care workers’ perceptions revealed a good fit, suggesting a one-dimensional solution (Tucker Lewis Index of factoring reliability=0.976; root mean square error of approximation index=0.079; 95% CI 0.063-0.096; Cronbach α=.88). Therefore, we calculated the scale’s mean score. To facilitate further analyses, we kept the coding of the 5-point Likert scale of the single item assessing the sufficiency of computers on the units.

All potential confounding and control variables, including facility and unit characteristics, perceptions of work environment factors, teamwork and safety climate, and care worker characteristics, are described in Multimedia Appendix 1.

### Data Analyses

Descriptive statistics (frequencies, percentages, means, and SDs) were calculated to describe the measured variables. To explore differences between care workers’ professional backgrounds with regard to the EHR system’s usefulness and whether a sufficient number of computers were available, we used chi-square tests. To explore the relationship between care workers’ perceptions with regard to the EHR systems and whether sufficient computers were available and implicit rationing of nursing care documentation, 2-level generalized linear mixed models were used. On the basis of the intraclass correlation coefficient 1 (ICC1), which was >0.05, multilevel modeling was required [34]. Therefore, we computed ICC1 to assess the variability of the outcome variable (implicit rationing of nursing care documentation) between units and facilities. In this case, an ICC1 of 0.155 at the unit level and 0.118 at the facility level indicated a need to account for the clustering of care worker data within units and facilities.

We report unadjusted (*crude*) associations and 2 adjusted models: (1) not including staffing and resources adequacy and (2) including staffing and resources adequacy. To compare the models’ relative fits, we used Akaike information criterion; a lower value indicates a better fit. Data analyses were performed with R (version 3.4.2; R Foundation for Statistical Computing, 2017) using the rptR package for the calculation of ICC1 [35] and the lme4 package for generalized linear mixed models [36]. Depending on the variable, between 0.1% and 8.3% of the data for unit and facility characteristics were missing. In the nurse survey, data missing varied between 0.1% (ie, educational background) and 3% (ie, professional experience). A *P* value of less than .05 was considered significant.

## Results

### Sample Description

This substudy used a sample of 1975 care workers. More than 90% were female; the majority were older than 41 years and had more than 5 years of professional experience. The majority worked part time, with employment levels between 51% and 90% and with regular changes in shifts. Of the 107 Swiss NHs included in the study, the majority were medium sized (between 50 and 100 beds) and private or privately subsidized. Table 1 summarizes the care worker, unit, and facility characteristics.

**Table 1:** Facility, unit, and care worker characteristics.

Facility and unit characteristics			Total (N=107 NHs^a^, 302 units, 1975 care workers)	German-speaking region (n=88 NHs, 268 units, 1794 care workers)	French-speaking region (n=19 NHs, 34 units, 181 care workers)
**NH size, n (%)**					
	Small (<50 beds)		24 (22.4)	20 (22.7)	4 (21.1)
	Medium (50-100 beds)		55 (51.4)	42 (47.8)	13 (68.4)
	Large (>100 beds)		28 (26.2)	26 (29.5)	2 (10.5)
**NH profit status, n (%)**					
	Public		45 (42.1)	41 (46.6)	4 (21.1)
	Privately subsidized or private		62 (57.9)	47 (53.4)	15 (78.9)
**NH unit characteristics**					
	Clinical focus on dementia, n (%)		218 (74.4)	196 (75.1)	22 (68.8)
	Bed capacity, median (IQR)		24 (12)	24 (12)	29 (19)
	Full-time equivalent per 100 beds, median (IQR)		48.5 (23.2)	48.0 (23.4)	51.6 (16.8)
	Skill mix level (% registered nurse), median (IQR)		26.5 (16.7)	27.8 (17.0)	20.3 (9.2)
**Care worker characteristics**					
	**Age (years), n (%)**				
		<21	127 (6.46)	120 (6.73)	7 (3.87)
		21-30	408 (20.76)	361 (20.24)	47 (25.97)
		31-40	336 (17.10)	295 (16.54)	41 (22.65)
		41-50	396 (20.15)	360 (20.18)	36 (19.89)
		51-60	556 (28.30)	519 (29.09)	37 (20.44)
		>60	142 (7.23)	129 (7.23)	13 (7.18)
	Gender: female, n (%)		1783 (91.25)	1613 (90.92)	170 (94.44)
	**Educational background, n (%)**				
		Registered nurse	944 (47.80)	861 (47.99)	83 (45.86)
		Licensed practical nurse	1031 (52.20)	933 (52.01)	98 (54.14)
	**Tenure in current nursing home, n (%)**				
		Up to 5 years	921 (48.02)	836 (47.96)	85 (48.57)
		5-10 years	387 (20.18)	348 (19.97)	39 (22.29)
		≥10 years	610 (31.80)	559 (32.07)	51 (29.14)
	**Employment level, n (%)**				
		<51%	319 (16.32)	303 (17.07)	16 (8.89)
		51%-90%	1105 (56.52)	982 (55.32)	123 (68.33)
		91%-100%	531 (27.16)	490 (27.61)	41 (22.78)
	**Main shift, n (%)**				
		Regular change of shifts	1003 (50.99)	921 (51.57)	82 (45.30)
		Day evening shift	783 (39.81)	702 (39.31)	81 (44.75)
		Night shift	181 (9.20)	163 (9.12)	18 (9.95)

^a^NH: nursing home.

### Variable Result Description

#### Care Workers’ Perceptions of the EHR System’s Usefulness and the Sufficiency of the Number of Computers on Their Unit

Overall, the care workers perceived their facilities’ EHR systems as useful (Table 2). The percentage agreeing or strongly agreeing with the respective statements ranged from 69.42% (*guarantees safe care and treatment*) to 78.32% (*allows quick access to relevant information on the residents*). However, less than half (46.61%) of the care workers reported sufficient computers on their units to allow timely documentation.

As summarized in Table 2, we observed differences between RNs’ and licensed practical nurses’ perceptions as well as between language regions. For instance, compared with RNs, licensed practical nurses more often agreed that *the EHR system gives a good daily overview of all residents on the care unit*.

**Table 2:** Care workers’ perception of the electronic health record system’s usefulness and of whether the number of computers was sufficient (N=1975).

6 items on care workers’ perceptions of the electronic health record system’s usefulness and sufficiency of the number of computers on the units	Total (N=1975), n (%^a^)	Educational background, n (%^a^)		*P* value^b^
		Registered nurses (n=966)	Licensed practical nurses (n=1058)	
The electronic health record system allows timely communication between the nursing and therapy teams	1367 (69.96)	667 (71.11)	700 (68.90)	.29
The electronic health record system provides a good overview on the main focus of care and treatment for the individual residents	1507 (76.89)	710 (75.53)	797 (78.14)	.17
The electronic health record system gives a good daily overview on all residents on the care unit	1429 (72.98)	664 (70.78)	765 (75.00)	*.04*
The electronic health record system guarantees safe care and treatment	1362 (69.42)	645 (68.54)	717 (70.23)	.42
The electronic health record system allows quick access to relevant information on the residents	1535 (78.32)	720 (76.51)	815 (79.98)	.06
On our unit there are sufficient computers to allow timely documentation	914 (46.61)	451 (47.98)	463 (45.35)	.24

^a^Percentage agreement (agree and strongly agree).

^b^Chi-square test, *P*<.05 highlighted in italic.

#### Implicit Rationing of Nursing Care Documentation, Work Environment, and Teamwork and Safety Climate

Approximately half of the care workers responded that they sometimes or often had to ration care activities related to documentation (range: 46.02% [*studying care plans*] to 50.06% [*set up or update residents’ care plans*]; Table 3). The mean rating for implicit rationing of nursing care documentation was 2.38 (SD 0.90; rarely to sometimes). As Table 4 shows, care workers rated adequate staffing and resources at the neutral midpoint (mean 2.67, SD 0.67) and strongly felt that they were supported by leadership (mean 3.18, SD 0.62). The mean teamwork and safety climate was rated as favorable (mean 3.89, SD 0.81). Furthermore, ICCs of the rationing of documentation items and whether sufficient numbers of computers were available ranged between 0.077 and 0.221, indicating substantial variation between units and between facilities (Table 4).

**Table 3:** Frequencies of implicit rationing of nursing care documentation (N=1975).

Care activities rationed by care workers in the last 7 days	Activity not necessary, n (%)	Never, n (%)	Seldom, n (%)	Sometimes, n (%)	Often, n (%)
Studying care plans at the beginning of the shift	13 (0.67)	478 (24.72)	553 (28.59)	480 (24.82)	410 (21.2)
Set up or update residents’ care plans	110 (6.83)	270 (16.77)	424 (26.34)	481 (29.88)	325 (20.19)
Documentation of care	4 (0.21)	429 (22.26)	654 (33.94)	561 (29.11)	279 (14.48)

**Table 4:** Characteristics of variables under study (N=1975).

Variables		Mean (SD)	Facility level, ICC1^a^ (95% CI)	Unit level, ICC1 (95% CI)
Rationing of nursing care documentation		2.38 (0.9)	0.118 (0.076-0.165)	0.155 (0.111-0.202)
Care workers’ perception of the electronic health record system’s usefulness		3.86 (0.77)	0.077 (0.043-0.112)	0.097 (0.064-0.135)
Care workers’ perception of sufficient number of computers		3.13 (1.33)	0.116 (0.072-0.161)	0.221 (0.176-0.269)
**Work environment**				
	Leadership	3.18 (0.62)	0.156 (0.104-0.205)	0.278 (0.228-0.326)
	Staffing and resources adequacy	2.67 (0.67)	0.214 (0.151-0.271)	0.254 (0.207-0.302)
Teamwork and safety climate		3.89 (0.81)	0.111 (0.068-0.156)	0.196 (0.152-0.244)

^a^ICC1: intraclass correlation coefficient 1.

#### Factors Associated With Implicit Rationing of Nursing Care Documentation

In the crude models (Table 5), as well as models 1 and 2 (Table 6), care workers’ perceptions of both the EHR system’s usefulness and whether a sufficient number of computers were available were significantly associated with implicit rationing of nursing care documentation. More positive care workers’ perceptions of the EHR system’s usefulness (β=−.12; 95% CI −0.17 to −0.06) and of the sufficiency of the number of computers (β=−.09; 95% CI −0.12 to −0.06) were associated with lower implicit rationing of nursing care documentation (model 2).

**Table 5:** Implicit rationing of nursing care documentation regressed on care workers’ perceptions of their electronic health record systems and the sufficiency of the number of computers, along with facility, unit and care worker characteristics and staffing variables, work environment, and teamwork and safety climate.

Variables			Crude models^a^	
			β (95% CI)	SE
**Explanatory variables**				
	Care workers’ perception of the electronic health record system’s usefulness		−.31^b^ (−0.36 to −0.26)	0.03
	Care workers’ perception of whether sufficient numbers of computers were available on their units		−.19^b^ (−0.21 to −0.16)	0.02
**Control variables**				
** **	**Facility characteristics**			
		Language region	0.18 (−0.03 to 0.40)	0.11
		Nursing home size	−.03 (−0.14 to 0.08)	0.05
		Profit status	−.04 (−0.19 to 0.12)	0.08
		Electronic health record system	0.01 (−0.01-to 0.04)	0.01
	**Unit characteristics**			
		Staffing levels	0 (−0.01 to 0.00)	0
		Skill mix levels	0 (−0.01 to 0.00)	0
	**Work environment**			
		Leadership	−.37^b^ (−0.44 to −0.31)	0.03
		Staffing and resources adequacy	−.63^b^ (−0.69 to −0.58)	0.03
	Safety and teamwork climate		−.39^b^ (−0.46 to −0.34)	0.03
	**Care workers’ characteristics**			
		Gender	−.07 (−0.21 to 0.06)	0.07
		Age	0.01 (−0.02 to 0.03)	0.01
		Educational background	−.08^b^ (−0.16 to −0.01)	0.04
		Professional experience	0.04 (−0.01 to 0.08)	0.02
		Employment level	−.04 (−0.09 to 0.03)	0.03
	Fixed effects (intercept)		2.39^b^ (2.32 to 2.47)	0.03

^a^Random effect: Facility-level variance (SD)=0.07 (0.27), Unit-level variance (SD)=0.06 (0.25).

^b^*P* value less than .05.

Higher ratings of leadership and safety teamwork climate were significantly associated with lower levels of implicit rationing of nursing care documentation only in model 1 (not accounting for staffing and resource adequacy). In model 2, care worker–perceived staffing and resources adequacy was the strongest explanatory factor, that is, higher ratings for staffing and resources adequacy were associated with lower levels of implicit rationing of nursing care documentation (β=−.52; 95% CI −0.58 to −0.45). Moreover, care workers’ educational backgrounds were significantly associated with implicit rationing of nursing care documentation in both models (Table 6), with licensed practical nurses in both cases reporting lower levels of rationing of nursing care documentation than RNs (β=−.09; 95% CI −0.15 to −0.02).

**Table 6:** Implicit rationing of nursing care documentation regressed on care workers’ perceptions of their electronic health record systems and the sufficiency of the number of computers, along with facility, unit and care worker characteristics and staffing variables, work environment, and teamwork and safety climate.

Variables			Multiple model 1^a^ (without staffing and resources adequacy)		Multiple model 2^a^ (with staffing and resources adequacy)	
			β (95% CI)	SE	β (95% CI)	SE
**Explanatory variables**						
	Care workers’ perception of the EHR^b^ system’s usefulness		−.14^c^ (−0.20 to −0.09)	0.03	−.12^c^ (−0.17 to −0.06)	0.03
	Care workers’ perception of whether sufficient numbers of computers were available on their units		−.12^c^ (−0.15 to −0.09)	0.02	−.09^c^ (−0.12 to −0.06)	0.01
**Control variables**						
	**Facility characteristics**					
		Language region	—^d^	—	—	—
		Nursing home size	—	—	—	—
		Profit status	—	—	—	—
		EHR system	—	—	—	—
	**Unit characteristics**					
		Staffing levels	—	—	—	—
		Skill mix levels	—	—	—	—
	**Work environment**					
		Leadership	−.12^c^ (−0.21 to −0.04)	0.04	0.08 (−0.04 to 0.12)	0.04
		Staffing and resources adequacy	—	—	−.52^c^ (−0.58 to −0.45)	0.03
	Safety and teamwork climate		−.20^c^ (−0.27 to −0.12)	0.04	−.08^c^ (−0.15 to −0.01)	0.04
	**Care workers’ characteristics**					
		Gender	—	—	—	—
		Age	—	—	—	—
		Educational background	−.08^c^ (−0.16 to −0.02)	0.04	−.09^c^ (−0.15 to −0.02)	0.04
		Professional experience	—	—	—	—
		Employment level	—	—	—	—
	Fixed effects (intercept)		4.67^c^ (4.39 to 4.94)	0.14	4.80^c^ (4.53 to 5.05)	0.13

^a^Random effects: Multiple model 1: Facility-level variance (SD)=0.05 (0.22), Unit-level variance (SD)=0.04 (0.21), Akaike information criterion=4598.8; Multiple model 2: Facility-level variance (SD)=0.03 (0.17), Unit-level variance (SD)=0.01 (0.12), Akaike information criterion=4405.8.

^b^EHR: electronic health record.

^c^*P* value <.05.

^d^Variable not included in the model.

## Discussion

### Principal Findings

In this study, we aimed to explore Swiss NH care workers’ perceptions of their EHR systems’ usefulness, whether their units had sufficient numbers of computers, and the association with rationing of nursing care documentation. Overall, the majority of care workers perceived the EHR systems as useful; however, fewer than half of the care workers reported having sufficient computers on their unit to allow timely documentation, and more than half of the care workers reported sometimes or often having to ration care activities related to documentation. Higher implicit rationing of nursing care documentation was reported by those who rated their EHR system’s usefulness as low and the number of computers as insufficient.

Most care workers in our study sample perceived that the EHR was useful, for example, that it provided a good overview of the main focus of care and treatment and allowed quick access to relevant information on residents. Earlier studies have found that various advantages of EHR compared with traditional paper records were reported in long-term care settings. These included the structured collection of and accessibility to information about residents’ family histories, contact information, medications, information regarding current and previous care, medical treatments and procedures, and other relevant health-related information [37]. Likewise, Swiss care workers appreciated the various benefits of their EHR systems. Although EHRs are supposed to improve the safety and quality of care by offering tools (eg, alerts and reminders) to help avoid adverse events such as those related to medication errors [8-12], nearly one-third of our sample did not consider the EHR useful for guaranteeing safe care and treatment. We cannot explain this perception, but it could be based on the structure, accessibility, monitoring tools, usability, or other aspects of EHRs as well as on the handling and common understanding of a team about how to deal with the system.

It is clear, however, that EHR use does not automatically improve documentation, that is, its adoption does not necessarily mean that its users will provide timelier, more complete records; better continuity of care; or safer care or treatments [38]. Although safety concerns linked to EHR implementation, especially during the initial adjustment to digital documentation, have been reported elsewhere [39], once care workers are familiar with their particular systems [40], EHRs ultimately have a strong potential to improve the quality and safety of workflows. As with other systems that have delivered widespread improvements, the expected benefits of EHR can only be achieved in real-world settings through continuous feedback and improvement [41]. Improving our understanding of how EHRs contribute to safe care and how their use in NHs may actually lead to safety issues will require further qualitative research.

One less complicated matter is that half of our respondents reported not having sufficient computers on their units for the timely completion of their documentation. Care workers, especially RNs, spend a considerable amount of their working time on documentation activities, such as developing or updating nursing care plans [19]. A lack of computers on the unit (often there is only one) might impede timely care planning and documentation and increase the documentation burden. Therefore, NHs need to allow care workers timely access to EHRs and avoid waiting times. For example, to eliminate waiting time for computers, it may be practical to perform activities such as developing or updating nursing care plans or documenting nursing care in real time at the patient’s bedside via mobile devices (eg, tablets or smartphones). Currently, however, no evidence is available on the effects or acceptability of such devices by NH care workers to either improve documentation or to reduce rationing of nursing care documentation. Further research on this topic is required.

More than half of our care worker sample responded that they sometimes or often had to ration documentation-related care activities. Tasks such as developing or updating nursing care plans or documenting nursing care are important parts of daily patient care; however, they are often perceived as keeping care workers away from the residents. However, it might be some time before EHR technology can meet care workers’ initial expectations that EHR use will reduce their documentation time, allowing them more time for direct care activities.

In fact, initial adjustment to EHR may even increase documentation time [18]. Although health care is a complex, adaptive system, the software is not. It is complex, but adaptation tends to result from incremental and iterative improvements. Initially, this limitation might be the heart of the problem for NH care workers: rather than following and lightening their daily workload, they might find that EHR largely determines and adds to it [42].

After adjusting for important factors, our analysis showed that rationing of nursing documentation is consistently related to care workers’ perceptions of both their EHR systems’ usefulness and the sufficiency of the number of computers available to them. This finding provides new insights on why these *indirect care* activities often remain unfinished [21,22]. Former evidence has shown that work environment factors such as leadership and staffing and resources adequacy as well as the safety and teamwork climate explain certain levels of NH care rationing [21,43]. In addition, we now see that both EHRs’ general lack of user-friendliness and the general unit-level shortage of documentation workstations are important factors explaining care workers’ tendency to leave *indirect* care activities, such as developing or updating nursing care plans or documenting nursing care, unfinished.

As this leaves information gaps in the EHR, documentation rationing is likely accompanied by work-arounds, such as exchanging vital daily information on paper and via oral handovers to provide continuity of care. In other situations, information may simply be lost. Apart from presenting obvious legal problems if documentation is lacking or untraceable, both options increase the risk of adverse events and reduce the quality of care.

In our study sample alone, we found 12 separate EHR systems, which might differ regarding key EHR domains (eg, data transfer, structured clinical documentation, medication use processes, and communication) [44]. EHRs target a large and growing global market; according to a recently published report from Fortune Business Insights, a compound annual growth rate of 5.4% is expected until 2026 [45]. As buyers in that market, NH management could more forcefully demand IT solutions that support care workers’ documentation needs while increasing safety and quality of care. EHR providers can reasonably be called upon to develop and design their software with input from all stakeholders—especially their users—in real-world settings. Therefore, care workers should be actively involved in testing and implementing the proposed IT infrastructure to ensure that, from the moment of implementation, it actually reduces their documentation burden [40].

### Limitations

First, the cross-sectional design of the study did not allow inference of causal relationships. Second, as both the outcome variable (rationing of nursing care documentation) and the main explanatory variables (both involving perceptions of IT infrastructure) were assessed via a care worker survey, this measure might have introduced common method bias. Third, we unfortunately did not measure when each NH implemented its EHR, what basic and/or continuous training care workers receive to use the EHR, or to what extent staff managers encourage or monitor the care workers in using the EHR information, which could have helped explain the association between care workers’ perceptions regarding IT infrastructure and implicit rationing of nursing care documentation.

### Conclusions

Although the surveyed RNs’ and licensed practical nurses’ overall perception of EHR systems’ usefulness in Swiss NHs was high, only half of the care workers reported having sufficient numbers of computers on their units. After adjusting for other main explanatory variables, our analyses indicated that more positive perceptions of both EHR systems’ usefulness and the sufficiency of the number of computers on their units were associated with less rationing of nursing care documentation. Thus, both the EHR system and the number of available computers influence care workers’ decision to leave *indirect* care activities, such as developing or updating nursing care plans or documenting nursing care, unfinished. Bearing this in mind, NH managers should carefully select and implement their IT infrastructure with full engagement and according to the needs of the end users, that is, their care workers, as well as their residents. Although EHRs are increasingly implemented in NHs, there is still little evidence on how their use influences the safety and quality of NH care, including as it relates to efficiency. Future challenges to the research concerning EHR use in NHs are (1) to identify user-friendly designs and successful implementations of related IT infrastructure in NHs (eg, EHR access via mobile devices) and (2) to evaluate the impact of EHR implementation in NH settings not only on both direct and indirect care processes but also on resident and care worker outcomes.

## Appendix

Multimedia Appendix 1 Study variables.

## Abbreviations

EHR: electronic health record
ICC1: intraclass correlation coefficient 1
IT: information technology
NH: nursing home
RN: registered nurse
SHURP: Swiss Nursing Home Human Resources Project
