# Supplementary material for: Electronic Health Record Use in Swiss Nursing Homes and Its Association With Implicit Rationing of Nursing Care Documentation: Multicenter Cross-sectional Survey Study
Source: JMIR Med Inform. 2021 Mar 2;9(3):e22974. doi: 10.2196/22974 (PMC7967228; doi:10.2196/22974)
Supplement: Multimedia Appendix 1 [file medinform_v9i3e22974_app1.doc]

**Appendix 1**

Table with study variables

| **Variable name** | **Description** | **Measurement** |
| --- | --- | --- |
| ***Outcome variable*** |  |  |
| Implicit rationing of nursing care documentation | Mean score of the 3-item subscale of the Basel Extent of Rationing of Nursing Care-Nursing Home version (BERNCA-NH), assessing, e.g., setting-up of care plans, documentation of care. | 5-point Likert scale with the following response options: 0 (=activity was not necessary), 1 (=never), 2 (=seldom), 3 (=sometimes), or 4 (= often);  Cronbach’s α (in this study) = 0.77 |
| ***Explanatory variables*** |  |  |
| Care workers’ perception of their EHR systems' usefulness | Mean score for the five items, assessing, e.g., care workers’ perception about timely communication, access to and overview of resident information. | Five self-developed items on a 5-point Likert scale from 1 (= strongly disagree) to 5 (= strongly agree)  Cronbach’s α (in this study) = 0.88 |
| Sufficiency of computers | Single item on the availability of a sufficient number of computers on their unit. | Self-developed item on a 5-point Likert scale from 1 = strongly disagree to 5 = strongly agree |
| ***Control variables*** |  |  |
| Facility characteristics |  |  |
| Language region | NH in the German- or French-speaking region | 1 = German-speaking,  2 = French-speaking |
| NH size | The facility's size, based on the number of long-term beds | 1 = Small (< 50 beds),  2 = Medium (50-100 beds), 3 = Large (> 100 beds) |
| Profit status | The type of NH based on financing | 1 = Public,  2 = Private subsidized, or  3 = Private |
| EHR system in place | The EHR systems implemented in the Swiss NH | Dummy variable (1 = yes / 0 = no) for each of the 12 EHR systems in the sample |
| Unit characteristics |  |  |
| Staffing levels | Full-time equivalent (FTE) positions divided by number of beds, multiplied by 100. | Number |
| Skill mix levels | Percentage of all FTEs per unit who are registered nurses . | Number |
| Work environment |  |  |
| Leadership | 5-item “Nurse manager ability, leadership, and support of care workers” subscale of the Practice Environment Scale–Nursing Work Index (PES–NWI), assessing direct supervisors in terms of the support they provided, their competency, back-up in decision-making, praise and recognition given, and the use of mistakes as learning opportunities rather than criticism. | 4-point Likert scale from 1 = strongly disagree to 4 = strongly agree  Cronbach’s α (in this study) = 0.86 |
| Staffing and resources adequacy | 3-item subscale “Staffing and resources adequacy” of the PES-NWI, assessing whether there was enough time and opportunity to discuss resident care problems, enough qualified personnel to provide quality resident care, and enough staff to get the work done. | 4-point Likert scale ranging from 1 (= strongly disagree) to 4 (= strongly agree);  Cronbach’s α (in this study) = 0.75 |
| Safety and teamwork climate | One 10-item single factor subascale for Teamwork and Safety Climate based on the Safety Attitudes Questionnaire, assessing opportunities to speak up or to ask questions when something is not understood, the extent to which other team members provide assistance when needed, the opportunity to discuss errors and to learn from each other, and the reception of feedback about one’s performance. | 5-point Likert scale from 1 (= strongly disagree) to 5 (= strongly agree), including the option “don’t know”  Cronbach’s α (in this study) = 0.96 |
| Care workers’ socio-demographic/professional characteristics |  |  |
| Gender | Care worker gender | 1 = female,  2 = male |
| Age | Care worker age in years | Years (in six categories) |
| Educational background | Care worker professional education | 1 = registered nurse,  2 = licensed practical nurse |
| Professional experience | Care worker professional experience | Years (in three categories) |
| Employment level | Care worker employment level | % employment (10-100) |
